# Topics of Public Interest

**Published:** 1918-01

**Authors:** 


### TOPICS OF PUBLIC INTEREST

**Coat of Arms of Medical Corps.** Col. C. C. McCulloch, Jr., M. D., Librarian, Surgeon-General's Library (Military Surgeon, Aug.) gives the technical heraldic description as follows:

Arms.—Per pale: dexter an escutcheon paly, of seven gules and six argent; chief dexter azure charged with mullets, five fessways and four paleways, argent; sinister argent charged with a serpent entwined on a staff paleways, all proper.

Crest.—A cock walking, gardant sinister, proper.

Motto.—Experientia et progressus.

In other words, the conventional shield bears on the longitudinal half at the viewer's left, a conventional U. S. flag with 20 stars, in four horizontal rows of five each, at the top, while the lower two-thirds contains longitudinally, the 7 red and 6 white stripes, the latter, like the right half of the shield being in silver. The right half bears a single-serpent caduceus. The shield is surmounted with a rooster, headed to the left but with the face turned toward the tail and a scroll below bears the motto. Strangely enough, there is no historic record of the adoption of the coat of arms but the number of stars (20) would date it between the admission of the 20th state, Mississippi, Dec. 10, 1817 and of the 21st, Illinois, Dec. 3, 1818, and this assumption is corroborated by the fact that the Medical Corps was organized by act of congress, April 14, 1818, from the previously existing Medical Dept. which, indeed, dated back to the Hospital Dept. organized in 1775 under Dr. Benjamin Church as Director General and Chief Physician.

---

**A New Specialty and a New Word Needed.** Orthopaedics, often written orthopedics and, perhaps on that account, perhaps because of the inevitable inclusion of foot deformities, understood to allude to the feet instead of to the straightening out of children generally, has come to have a new and far different meaning since the beginning of the present war. The correction of traumatic deformities, the restoration of function of joints and muscles, the correction of inevitable cripplings by artificial devices, the education of more or less handicapped soldiers in manual and other muscular movements in the various arts and crafts, obviously constitute a specialty involving quite different principles and methods from orthopaedics in the old sense, though depending upon the same experience and general kind of therapeutic skill. But, while this new specialty necessarily draws at present upon the personnel of orthopaedists, it is doubtful whether the two lines of work will not practically demand a division of fields, in the future, perhaps as soon as a sufficient number of men have been trained in the new specialty. Here is need of a new term and we miss the guidance of the late Dr. Achilles Rose. Tentatively, we suggest Orthandrics or Orthostratics (man or soldier—straightening).

---

**Educational Value of Patent Medicine.** The Medical Economist quotes the following advertisement from an Ohio paper. The therapeutic effect is not more wonderful than many others reported from patent medicines but the incidental educational value is very modestly overlooked by the manufacturers. They should have their product adopted by the public schools.

"I took the whooping cough when I was only two months old. It settled on my lungs and I gradually declined until it was necessary to carry me on a pillow. They took me to the doctor and tried every one they thought would help me, but got little relief from my cough. After taking a bottle of your medicine I found that I was improving very much. I have taken Nacor until my cough is almost gone. I am now seven years old, weigh 46 pounds and go to school every day. I give Nacor all the praise for my relief and hope that I can only cause someone else to try it.''

---

**Missing Links of Buffalo Medical Journal Wanted.** Vol. 15 of the old series was published in N. Y., under the editorship of Dr. Austin Flint, Jr., under the title of New York Monthly Review and Buffalo Medical Journal, the first number being that of June 1859. The twelfth number mentions an extra issue containing lectures of Prof. Dalton, supplementary to April issue of the 13th volume, and a thirteenth, June issue with title page and index, lacking from our file of bound volumes. The twelfth (May 1860) issue goes on to state that ''with the succeeding or July number, will commence the first number of the combined journal'' but we find no explanation of what is meant by this term. We understand, however, that the old series was merged with the American Medical Monthly in July 1860, but have no copies of this combined journal. The new series was begun in August 1861, the editor modestly refraining from giving his name—unless on the cover which is absent from our bound file—but seems to have been Dr. Miner, from a letter published. The title was Buffalo Medical and Surgical Journal and Reporter, explained in a sarcastic note at the end of the first issue, as due to the prohibition of the original name by Mr. A. I. Mathews (sic), who was a druggist well known to old Buffalonians and who had some property rights in the old series. From this time on, our file is complete. The exact title was changed several times and, under the management of Dr. Potter, reverted to the original form and the numbering of volumes was made continuous from the beginning of the old series. We hope that some of our readers can supply the missing links mentioned and furnish a historic article on the editorial changes of the Journal.

---

**Pay of Army Surgeons for the Civil War.** Our issue of Nov. 1861 quotes from a circular from the War Dept., Jan. 1860 as follows: Asst. Surgeon, under five years' service $53.33 per month, rations $36, forage for horse $8, servant $12, clothing for servant $2.50, servant's rations $9. Total receivable $120.83. Over five years' service, $70 per month, other allowances same, total receivable $137.50. Total receivable over ten years' service, $173.50. Surgeon, under ten years' service, $80 per month, total amount for servant $47, aggregate receivable, including horse and servant, $187. Surgeon over ten years' service, $80, aggregate amount receivable $223. Allowance for forage and servants is paid only when they actually employ and keep in service the number of servants and horses charged for. In addition, both assistant and full surgeons are allowed an additional ration per day after the termination of every five years' service. (Note: As we understand the terms, the actual receipts besides allowances for horse and servant were as follows: 1st five years, assistant surgeon, $1072. 2d five years without promotion, $1704. 2d five years with promotion to grade of surgeon, $1824. 3d five years, rank of surgeon, $2256. 4th five years, rank of surgeon, $2688. Considering the different value of the dollar in purchasing power, these salaries are not very different from the present).

In January, 1862, a bill was introduced providing for a Surgeon-General, ranking as Brigadier General, a Sanitary Inspector General ranking as Colonel, six Sanitary Inspectors ranking as Lt. Colonels, Surgeons of first class ranking as majors, 50 Surgeons of second class ranking as captains and not over 70 Assistant Surgeons ranking as 1st lieutenant, all as of cavalry. This was essentially the same as the system up to the present, except as to the numbers. Salaries are not mentioned but editorially, the bill is highly commended and they were therefore, apparently regarded as satisfactory.

---

**Patriotism and Common Sense.** In spite of the opposition to the teaching of German in the public schools, it is interesting to note that more pupils have elected this study in Buffalo than before. If there ever was a time when loyal Americans needed to know German, this is it. In this connection, it may be said that the expression of prejudice against everything German is of very little military value. To Americans of German descent who may have considered a change of name, we would point out the confusion which would have resulted if, at the time of the Revolution, British surnames had been discarded for those of indigenous origin. So far as we have ever heard, nothing of the sort was even suggested. The President has made it clear that we bear no malice toward the German people, let us not imagine that we are showing loyalty by trying to annihilate either the German language, German science, especially medical, or German music—altogether it is obviously in bad taste under present conditions to play the national airs of hostile nations. We understand that a woman has been arrested for criticising the uniform of our soldiers. Hence, if called to service, learn to retract your larynx and remember that the priority of breeches over shoes in dressing is obligate and not faculative as in peace. But, we will take the chance of being hanged by remarking that it is not edifying, either aesthetically or as indicative of our commercial independence in regard to the dye industry to see a squad of eight men attired in sixteen shades of clothing from something properly termed olive drab, through various brindles down to a soiled cream color.

---

**Unit of 100 American Army Surgeons Organized for Service in the Hospitals of Roumania.** This is the first detachment under the Surgeon-General to be sent for service abroad other than with the French, British or American forces. This detachment is under Col. Walter D. McCaw of the Regular Army and in addition to the 100 Army Surgeons are a few nurses.

---

**Tuberculosis in War.** It is stated that half a million cases of tuberculosis have developed in the French Army—about 1/8 of the entire number of troops—and that, while statistics are not available, the disease is rife among the Germans. About 2% of the men called for draft examination in this country show active lesions. Against the cost of war must be credited the benefit of knowing where we stand in the matter of tuberculosis, possibly the benefit of relief of incipient cases by camp training. On the other hand, an additional charge against war will be the spread of infection in active service under unfavorable hygienic and sanitary conditions.

---

**Wanted: Pets for the Army.** The boys in the camps of the new National Army are sending out requests for pets of any variety.

---

**Health at Camps Improve.** Pneumonia epidemics in some southern camps, most prevalent where there are many cases of measles. The sick rate in the National Guard is 42.6 a thousand, compared with 28.5 for the National Army men. The highest rate come from camps at which southern troops are training.

---

**Report of Health Conditions Among the American Soldiers in France.** Percentages given are on the basis of 1,000 men per year. Pneumonia, 16.6; dysentery, 2; malaria, 1; venereal diseases, 181.5; typhoid, 0; para-typhoid, 0; measles, 21.7; meningitis, 1; scarlet fever, 1.9 per cent.

**Hospital Ready at Fort Niagara.** Twelve new hospital buildings with wide verandas are ready for occupancy. They have a capacity of about 300. A base hospital probably will be established there as soon as there is need for it.

---

**Base Hospital Unit No. 23,** organized and recruited in Buffalo, left November 21st, for somewhere presumably France. It will undoubtedly be the first Buffalo unit to cross the ocean.

---

**Health Commissioner Fronczak** does not approve of housing persons in old freight cars as a means of meeting the shortage of living accommodations in Buffalo. He is trying to enlist the aid of a philanthropist to build model tenements.

---

**Buffalo Medical Station Meets Argument.** Health Commissioner Fronczak recommends that a public station be established under the direction of the department of health for advice, prevention and early treatment of venereal diseases. He would have the station in the district bounded by Main, Pine, Exchange and Genesee Streets. Dr. Walter S. Goodale, superintendent of hospitals and dispensaries, while not opposing the purpose of the station, questioned its advisability, especially as the city is adequately equipped with dispensaries, health centers and with a hospital where such diseases may be treated. He said that the station would have to have a sign indicating its purpose and the district in which it would be located would receive a certain stigma thereby. He doubted whether persons needing treatment would go to such a place. Dr. Fronczak and Dr. Grover Wende said that the station should be near the source of infection. Dr. Wende said that there is such a station at each army cantonment and that the war department has recommended them for cities for the protection of soldiers.

---

**Medical Staff for Home Defense Corps.** Dr. Earl P. Lothrop will be major in charge of the hospital corps; Dr. A. H. Cooke will be captain and surgeon of the 2d battalion; Dr. Lawrence Hendee will be surgeon of the 3d battalion with rank of captain.

---

**The Universal Military Training League,** 1st National Bank Bldg., Chicago, is seeking to secure legislation now that would authorize universal military training as soon after the cantonments were vacated, as the War Department can handle the matter without embarrassment in the conduct of the war. It feels that if we wait until the war is ended, questions of peace and readjustment will so obsess the people that military training will be side-tracked and no thought will be given to keeping ourselves in a condition for future defense.

---

**The Buffalo Marine Hospital,** which formerly accepted sailors only, is now open to all branches of the service and to all federal employees.

---

**The J. N. Adam Memorial Hospital at** Perrysburg has been granted $15,000 by the councilmen to be used for the erection of new buildings.

---

**Scarlet Fever Serum.** Dr. Carl Klin, a bacteriologist and an assistant physician in charge of the Stockholm contagious hospital, has announced to the Swedish Medical Society the discovery of a serum for scarlet fever. He said that the serum had reduced the mortality in the severest cases to 17 3-5% against more than 70% in equally severe cases which were not treated with the serum. Blood was taken from convalescent patients and was allowed to stand until the serum had seperated from the other blood constituents. This was administered intravenously by the use of 20 cubic centiliters for small children, and up to 50 or 60 for adults. This treatment had no effect on nephritis.

---

**Automobiles** caused the death of 801 persons in New York State during the first ten months of 1917, an increase of 197 over the figures for the corresponding period of 1916.

---

**Smallpox** has developed in two negroes working for the Lehigh Valley Railroad. All of the 100 negroes who are quartered by the company in railway cars have been vaccinated by order of the Health Commissioner and a guard has been placed around the cars.

---

**Births and Deaths.** Health Officer W. A. Scott of Niagara Falls, has reported to City Manager O. E. Carr that statistics compiled by the bureau of vital statistics show that there were 45 deaths and 127 births in November.

---

**Deaths in Army Cantonments.** For the week of Nov. 23, 97 died out of 374,762 national guard soldiers and 60 out of 426,310 national army soldiers. Roughly speaking, men of military age would normally have a death rate of about 5:1,000 per annum or, say, 1:10,000 per week.

